# Progress of the National Insurance Bill

**Published:** 1911-07-29

**Authors:** 


### HOSPITALS AND STATE INSURANCE.

### PROGRESS OF THE NATIONAL INSURANCE BILL.

The political developments of the last few days and the attitude of the Labour party towards the National Insurance Bill have rendered the future of the measure somewhat uncertain. It is understood, however, that it is the present intention of the Government to proceed with the measure and to endeavour to reconcile conflicting interests. The position of hospitals under the proposed scheme will be discussed by the House at an early date, and Mr. Lloyd George has consented to consider favourably amendments framed with the object of improving the position of hospitals. When the Committee stage is resumed, Clause 12 will come under consideration. This clause provides that no payment shall be made in respect of sickness, etc., benefit during any period when an insured person is an inmate of "any workhouse, hospital, asylum, or infirmary, supported by any public authority or out of any public funds or by a charity, or of a sanatorium or similar institution." It also provides that if such person is an inmate of a sanatorium or similar institution and has no dependents, the benefit shall be paid to the Local Health Committee towards the general purposes thereof. Notice has been given of a number of amendments of this clause. Mr. Cassel will move an amendment providing that whilst an insured person is an inmate of a hospital or other institution, the benefit to which he is entitled shall be applied in part in paying to the proper authorities of any hospital or other charitable institution such sums as may have been agreed upon. Mr. Austen Chamberlain intends to move that if an insured person be an inmate or patient of a hospital or institution supported by voluntary subscriptions, the benefit shall be divided between that hospital and his dependents in such proportions as the Local Health Committee may determine. Mr. Hume-Williams has given notice of an amendment providing that the benefit in respect of an insured person who is an inmate of a hospital shall be paid to the funds of such hospital. An amendment stands in the name of Mr. Goulding that such benefit as is not paid to dependents of an inmate of a hospital, etc., shall be paid to the authorities of the hospital, etc., "towards the refunding of any expenditure actually incurred in respect to the insured person." Another amendment, of which notice has been given by Mr. Cassel, empowers Friendly Societies and health committees to enter into agreements with hospital authorities for the payment of any expenditure incurred by the hospital in providing medical treatment for an insured person. A number of new amendments to Clause 14 have been put down, including several by the Chancellor of the Exchequer, which are intended partially to meet the demands of the medical profession, and it is understood that others will be put down in the course of the next few days. Sir Henry Craik has given notice of an amendment providing that every person whose income from all sources exceeds £104 a year and has become entitled to medical benefit shall be entitled in lieu of medical attendance to receive an equivalent pecuniary allowance towards the expenses of medical treatment to be calculated in accordance with regulations made by the Insurance Commissioners, and any person whose income from all sources does not exceed £104 a year shall be entitled to elect in lieu of medical attendance to receive such equivalent pecuniary allowance as above mentioned. Replying to Mr. Evelyn Cecil, who asked the Chancellor of the Exchequer whether he could give any information as to the extent to which members of Friendly Societies had in the past had medical attendance at voluntary hospitals instead of, or in addition to that of, their club doctors, Mr. Hobhouse said no such information was available. Replying to a question asked by Mr. Houston, Mr. Hobhouse said the German sickness insurance societies had had their own disputes with the doctor, and as a result were now paying higher fees than formerly, but there was no evidence to show that the medical treatment was now unsatisfactory; no doubt the medical service was most efficient where the societies and the doctor were satisfied with the contracts under which it was given.

